# Effects and safety of repetitive transcranial magnetic stimulation on cognitive outcomes in mild cognitive impairment: a systematic review and meta-analysis

**DOI:** 10.3389/fnagi.2026.1894724

**Published:** 2026-09-24

**Authors:** Hua Ren, Jian Jiao, Xinru Lan, Chunyan Tan, Ziqi Wang, Haonan Pei, Dezhong Yao, Tiejun Liu, Li Dong

**Affiliations:** 1 The Clinical Hospital of Chengdu Brain Science Institute, MOE-K Lab for Neuroinformation, Brain-Apparatus Communication Institute, University of Electronic Science and Technology of China, Chengdu, China; 2 School of Life Sciences and Technology, University of Electronic Science and Technology of China, Chengdu, China; 3 The Fourth People’s Hospital of Chengdu, Chengdu, China; 4 Sleep Medicine Center, Mental Health Center, National Center for Mental Disorders, Sleep Research Laboratory, West China Hospital, Sichuan University, Chengdu, China; 5 Research Unit of NeuroInformation, Chinese Academy of Medical Sciences, Chengdu, China

**Keywords:** cognitive function, meta-analysis, mild cognitive impairment, randomized controlled trials, repetitive transcranial magnetic stimulation

## Abstract

**Background:**

Repetitive transcranial magnetic stimulation (rTMS) is a non-invasive neuromodulation technique that has been increasingly investigated as a potential treatment for mild cognitive impairment (MCI). However, its clinical efficacy remains uncertain due to heterogeneous findings across studies.

**Methods:**

A systematic review and meta-analysis of randomized controlled trials comparing active rTMS with sham stimulation or matched control interventions was conducted. Electronic databases were searched from inception. Eligible studies included patients with MCI and reported cognitive outcomes using validated scales, including the Montreal Cognitive Assessment (MoCA), Mini-Mental State Examination (MMSE), or Rivermead Behavioral Memory Test (RBMT).

**Results:**

Fourteen studies contributing 15 datasets were included. Compared with sham or control interventions, rTMS was associated with higher post-intervention scores on the MoCA, MMSE, and RBMT. For the primary MoCA outcome, the pooled effect estimate was significant but accompanied by substantial between-study heterogeneity (Hedges’ *g* = 1.46, 95% CI 0.97 to 1.94; *p* < 0.001; I^2^ = 86.0%). Exploratory subgroup analyses showed significant between-subgroup differences according to stimulation frequency (*p* = 0.021) and stimulation site (*p* < 0.001), whereas the difference according to risk of bias was not statistically significant (*p* = 0.114). Among the 10 datasets reporting quantitative safety outcomes, no significant increase in adverse events was observed compared with sham stimulation (RR = 1.25, 95% CI 0.76 to 2.04; *p* = 0.374).

**Conclusion:**

rTMS may improve cognitive test performance in patients with MCI. However, substantial between-study heterogeneity limits the precision and generalizability of the estimated treatment effect. Although exploratory subgroup analyses suggested variation according to stimulation frequency and site, these findings do not establish definitive effect modifiers or optimal stimulation parameters. Further well-designed, adequately powered trials with rigorous blinding, standardized protocols, and clinically meaningful outcomes are needed to clarify the magnitude and clinical relevance of rTMS effects in MCI.

## 1 Introduction

As global population ageing deepens, cognitive impairment has emerged as a major public-health concern that compromises the well-being of older adults and places an increasingly heavy burden on medical and social care systems (Yim and Moon, 2025). Cognitive impairment spans a continuum of decline from mild cognitive impairment (MCI) to overt dementia, characterized by deficits across multiple cognitive domains – most notably memory, executive function, attention and language (Duff, 2024; Mian et al., 2024). Such deficits substantially erode patients’ functional independence and quality of life, while imposing long-term caregiving pressure on families and on society at large (Shi et al., 2025). Timely identification of individuals with MCI and early intervention may delay or even halt the progression to dementia, representing a critical stage in dementia prevention and management (Zhao et al., 2022; Ding et al., 2023).

Currently, clinical interventions for cognitive impairment remain limited in scope. Pharmacological treatments offer only modest benefits, are indicated for narrow patient populations, and are frequently constrained by tolerability and long-term adherence concerns (Reuben et al., 2024). Conventional non-pharmacological interventions, although generally safe, are often criticized for their slow onset of action, variable adherence, and inconsistent effectiveness, and consequently fall short of the clinical demand for an efficient, safe and scalable cognitive intervention (Ding et al., 2023). Exploring novel interventions that are safe, non-invasive, well-tolerated, and capable of improving cognition and slowing disease progression has therefore become a priority research direction in the field of cognitive disorders (Caporlingua et al., 2025; Khachaturian et al., 2025).

Repetitive transcranial magnetic stimulation (rTMS) is a non-invasive neuromodulation technique that has been widely applied to a range of neurological and psychiatric disorders (Kan et al., 2023), owing to its ease of use, favorable safety profile and generally mild side-effect profile. A growing body of clinical research indicates that rTMS can meaningfully improve core cognitive domains – including memory, executive function and attention – in patients with cognitive impairment, thereby raising overall cognitive performance and providing a promising non-pharmacological therapeutic option (Liu et al., 2024; Ebrahimzadeh and Soltanian-Zadeh, 2026). Multiple primary trials and previous meta-analyses have reported that rTMS improves global cognitive function and activities of daily living to varying degrees, with demonstrable benefit across populations diverse in etiology and severity (Wang et al., 2021; Zhang X. et al., 2025).

Despite this encouraging signal, existing evidence is characterized by significant heterogeneity: stimulation protocols vary widely across studies, outcome-assessment frameworks lack standardization, and the resulting effect estimates are heterogeneous (Gao et al., 2023; Ong and Tang, 2025; Rektorová et al., 2025). In clinical practice, there is still no clear, evidence-based guidance on the overall efficacy of rTMS, the populations most likely to benefit, or the optimal stimulation parameters, making it difficult to support rational, standardized clinical decision-making (Antonioni et al., 2025; Fuseya et al., 2025). The present systematic review and meta-analysis therefore synthesizes the current randomized controlled evidence, with the twin aims of (i) quantifying the effect of rTMS on cognitive performance, with Montreal Cognitive Assessment (MoCA) scores as the primary outcome, and (ii) evaluating its clinical applicability and safety profile in patients with MCI.

## 2 Materials and methods

### 2.1 Protocol and reporting standards

This systematic review and meta-analysis was reported in accordance with the Preferred Reporting Items for Systematic Reviews and Meta-Analyses (PRISMA) 2020 statement (Figure 1; Page et al., 2021a,b). A comprehensive literature search was performed across both English- and Chinese-language electronic databases, including PubMed, Embase, Web of Science, CNKI and Wanfang Data, from database inception through March 27, 2025. Reference lists of eligible trials and previous relevant systematic reviews were hand-screened to identify additional records. The search combined MeSH terms and free-text keywords related to the intervention (“repetitive transcranial magnetic stimulation,” “rTMS,” “transcranial magnetic stimulation”) and the population (“cognitive impairment,” and “mild cognitive impairment”). No language restriction was applied at the search stage; language eligibility was assessed at the full-text screening stage.

**FIGURE 1:**
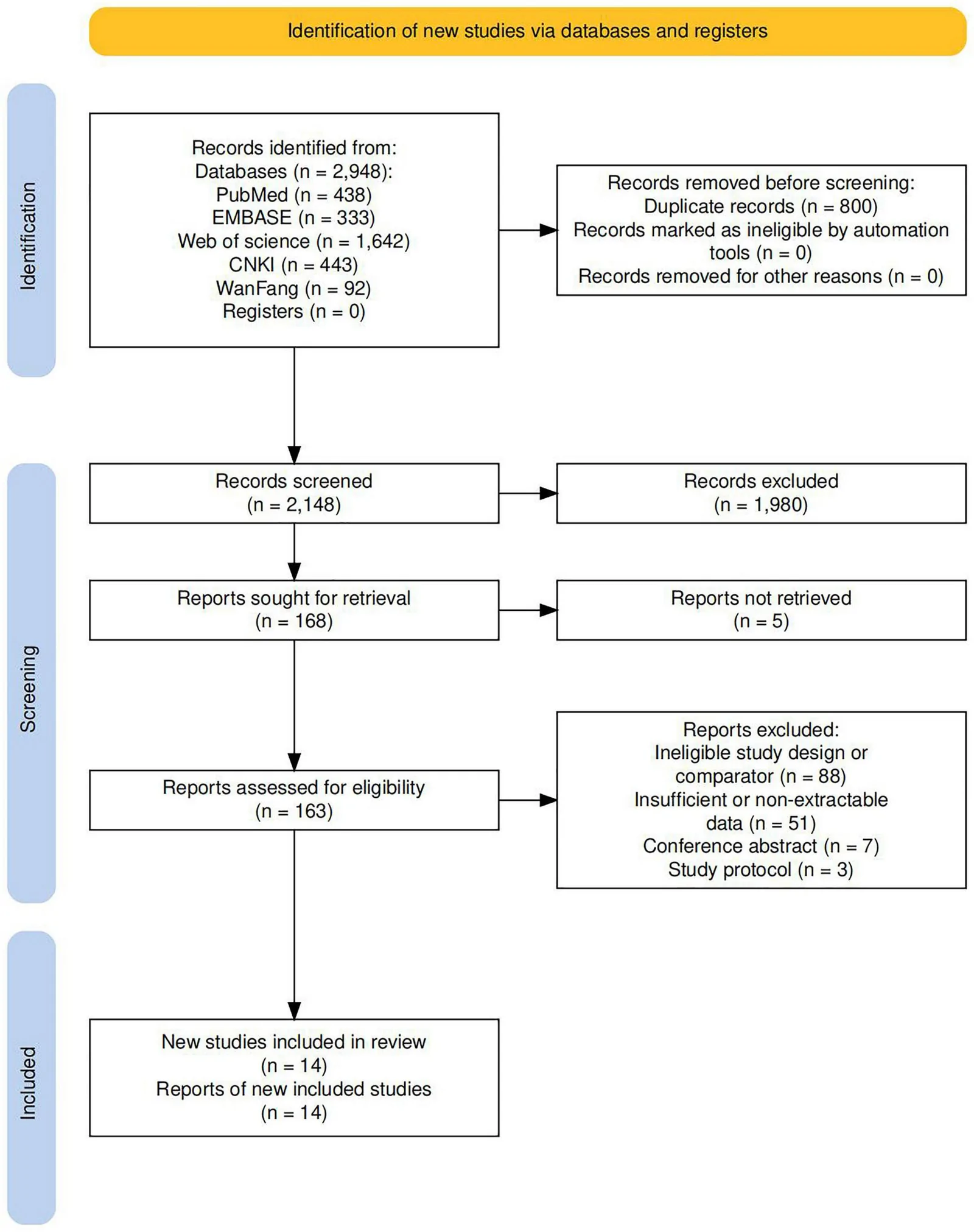
PRISMA 2020 flow diagram of the study identification, screening, eligibility assessment, and inclusion process. The diagram summarizes the identification, screening, eligibility assessment, and final inclusion of studies in the systematic review and meta-analysis.

### 2.2 Inclusion criteria for the selection of studies

Studies were included if they met all of the following criteria: (i) randomized controlled trials comparing active rTMS with a sham control or evaluating rTMS as an add-on intervention (e.g., rTMS plus co-intervention versus the same co-intervention alone); (ii) participants with clinical diagnosis of MCI established by recognized diagnostic criteria (e.g., Petersen criteria, DSM-5); (iii) reporting of at least one of the pre-specified cognitive outcomes [MoCA, Mini-Mental State Examination (MMSE), and Rivermead Behavioral Memory Test (RBMT)] at post-intervention assessment with extractable means, standard deviations and sample sizes; (iv) publication in English or Chinese. Studies were excluded if they were non-randomized, single-arm, or lacked an eligible comparator; used a region- or volume-of-interest neuroimaging design incompatible with the present outcome synthesis; reported samples that overlapped with an already-included trial; lacked extractable between-group outcome data; enrolled participants with comorbid Parkinson’s disease or other confounding neurological conditions; or were published in a language other than English or Chinese.

### 2.3 Study selection and data extraction

Two reviewers independently screened titles and abstracts against the eligibility criteria; subsequently, potentially relevant records underwent full-text assessment. Disagreements were resolved through discussion and, where necessary, adjudication by a third reviewer. A standardized data-extraction form was employed to extract the following data for each included trial: sample size per arm, participant characteristics, TMS protocol, outcome measures and time points, and reported adverse events. Data were cross-checked by a second reviewer and validated against the authoritative primary dataset maintained by the data custodian. For crossover trials (Gy et al., 2021), each treatment period was treated as a separate comparison. Because the within-subject correlation required to account for the paired design was unavailable, the two periods were analyzed as independent observations. This approach does not fully account for the dependence between repeated observations from the same participants and may therefore underestimate the variance of the pooled estimate.

### 2.4 Statistical analysis

#### 2.4.1 Effect-size calculation

All statistical analyses were performed using Stata/SE version 16.0 (StataCorp LLC, College Station, TX, USA). Treatment effects were estimated using post-intervention cognitive scores. For each study, effect sizes were calculated as the between-group difference in post-treatment means comparing the active rTMS group with the sham or control group for pre-specified outcomes, including the MoCA, MMSE, and RBMT. Continuous cognitive outcomes were synthesized using Hedges’ g with 95% confidence intervals. Hedges’ g was used as the standardized effect-size measure because it incorporates a correction for the upward bias of standardized mean differences in small samples. Because the primary analysis was based on post-intervention values, this approach assumes baseline comparability between groups through randomization. Potential baseline imbalances were considered when interpreting the findings. Positive effect sizes indicated better cognitive performance in the active rTMS group. For add-on intervention trials, the estimated effect size was interpreted as the incremental benefit of rTMS beyond the co-intervention.

#### 2.4.2 Data synthesis and heterogeneity

Effect sizes were pooled using a random-effects model. Heterogeneity was assessed using Cochran’s Q statistic and the I^2^ index, with I^2^ values of approximately 25%, 50%, and 75% interpreted as low, moderate, and high heterogeneity, respectively (Ioannidis, 2008).

#### 2.4.3 Publication bias

Publication bias and small-study effects were assessed using funnel plots and Egger’s regression test when at least 10 independent studies were available, with *p* < 0.05 indicating evidence of small-study effects (Egger et al., 1997). For outcomes with fewer than 10 studies, funnel plots were presented for descriptive purposes only. For the MoCA analysis, the crossover study by Gy et al. (2021) was excluded because its two comparisons were not statistically independent. When asymmetry or small-study effects were detected, trim-and-fill analysis was applied as a sensitivity analysis (Shi and Lin, 2019).

#### 2.4.4 Subgroup analyses

Seven subgroup analyses were performed on the primary MoCA outcome to explore potential sources of heterogeneity, stratified by: (i) overall risk of bias (low vs. high/unclear); (ii) stimulation modality (rTMS alone vs. rTMS combined with an adjunctive intervention); (iii) diagnostic criteria (Petersen vs. non-Petersen vs. unspecified); (iv) stimulation frequency (1 Hz vs. ≥ 5 Hz); (v) stimulation intensity [< 100% vs. ≥ 100% of resting motor threshold (RMT)]; (vi) cumulative pulse exposure (< 30,000 vs. ≥ 30,000 pulses); and (vii) stimulation site (left Dorsolateral Prefrontal Cortex (DLPFC) vs. bilateral DLPFC vs. right DLPFC vs. other). Each subgroup-specific pooled estimate was computed using the same random-effects model, and between-subgroup differences were tested using a fixed-effect Cochrane Q-test for interaction. Subgroup classifications were drawn directly from the pre-coded categorical variables curated in the primary data file to ensure consistency with the original trial-level coding.

#### 2.4.5 Meta-regression analyses

To further explore potential sources of heterogeneity in the primary MoCA analysis, exploratory univariable meta-regression analyses were conducted using 12 independent studies. Mean age, baseline MoCA score, number of treatment sessions, cumulative pulse exposure, and treatment duration were examined separately as continuous study-level moderators. Between-study variance was estimated using restricted maximum likelihood, with the Knapp–Hartung modification applied for statistical inference (Supplementary Table 1).

#### 2.4.6 Sensitivity analyses

Sensitivity analyses were conducted to assess the robustness of the primary MoCA findings. First, a leave-one-out analysis was performed by sequentially omitting each study and recalculating the pooled effect size to examine whether the overall results were driven by any single trial. Second, the stability of the pooled estimates was evaluated by comparing results obtained from the random-effects model with those from a fixed-effect model. Third, influence diagnostics were assessed using a Baujat plot to identify studies that contributed disproportionately to between-study heterogeneity and to the overall pooled effect. In addition, because the two treatment periods of the crossover trial (Gy et al., 2021) were analyzed as independent comparisons, a separate sensitivity analysis was performed by excluding both comparisons from this study and recalculating the primary MoCA pooled estimate.

#### 2.4.7 Adverse-event synthesis

For each trial reporting quantitative safety data, the number of participants experiencing at least one adverse event was extracted for the active and sham arms. A Mantel–Haenszel pooled risk ratio (RR) with 95% confidence interval was calculated across trials with non-zero events; trials reporting zero events in both arms were included using a 0.5 continuity correction where necessary. Reported event types, severity, attribution and impact on trial retention were additionally summarized narratively. Trials providing no usable quantitative safety information were explicitly flagged as contributing incomplete safety evidence.

### 2.5 Risk-of-bias assessment

The methodological quality of each included trial was evaluated using an adapted risk-of-bias framework based on the Cochrane Risk of Bias Tool (RoB 1.0). Six prespecified domains were assessed: random sequence generation, allocation concealment, blinding of participants and personnel, incomplete outcome data, selective outcome reporting, and other sources of bias (Ma et al., 2020). Each domain was assessed as having a low, unclear, or high risk of bias by two independent reviewers, with disagreements resolved by consensus. Domain-level and study-level judgements were summarized in Supplementary Table 2. Risk-of-bias assessments were also used to examine whether methodological quality was associated with variation in the estimated treatment effects, with trials categorized as having low versus high/unclear overall risk of bias for subgroup analysis.

## 3 Results

### 3.1 Study selection and characteristics

The study selection process is summarized in Figure 1. A total of 2,948 records were identified through database searching. After removing 800 duplicates, 2,148 records were screened by title and abstract, of which 1,980 were excluded. A total of 168 reports were sought for retrieval, and 163 full-text articles were assessed for eligibility after excluding 5 unretrieved reports. Of the 163 full-text reports assessed for eligibility, 149 were excluded: 88 did not use an eligible randomized controlled design or comparator, 51 did not provide sufficient or extractable between-group cognitive outcome data, 7 were conference abstracts without sufficient full-text information, and 3 were study protocols without outcome data. Finally, 14 studies were included in the meta-analysis, comprising 6 English-language and 8 Chinese-language publications (Han et al., 2013; Drumond Marra et al., 2015; Sun and Ma, 2015; Wen et al., 2018; Deng et al., 2019; Zhu et al., 2020; Gy et al., 2021; Zhang and Pan, 2021; Zhou and Chen, 2023; Lv et al., 2024; Chen et al., 2025; Liu et al., 2025; Quan et al., 2025; Riccitelli et al., 2025). One crossover trial was included as two independent comparisons, resulting in a total of 15 datasets in the quantitative synthesis (Gy et al., 2021). Fourteen datasets contributed to the primary MoCA analysis, five to MMSE, and three to RBMT. The included datasets comprised 706 participants (rTMS *n* = 367; sham *n* = 339). Participant characteristics and study details are summarized in Table 1, with stimulation parameters provided in Table 2. Participants were aged approximately 60–75 years and diagnosed with MCI. Most studies used Petersen criteria, while others applied alternative or unspecified diagnostic frameworks.

**TABLE 1:** Demographic and clinical characteristics of the included studies.

Study	Diagnosis criteria	Sample size		Gender (Male%)		Age, year (mean ± SD)		Education (mean ± SD)		MMSE (mean ± SD)		MoCA (mean ± SD)		Intervention		Outcome measure
		n1	n2	n1	n2	n1	n2	n1	n2	n1	n2	n1	n2	Inter vention	Control	
Zhu et al., 2020	Peterson	13	12	53.8	41.7	64.17 ± 5.21	65.91 ± 4.93	NA	NA	NA	NA	22.30 ± 1.36	21.82 ± 1.62	rTMS	Sham rTMS	Moca
Han et al., 2013	Peterson	22	18	36.4	33.3	66.5 ± 5.02	66.7 ± 5.25	11.35 ± 2.23	11.11 ± 3.16	NA	NA	22.85 ± 2.21	23.33 ± 2.35	rTMS	Sham rTMS	Moca
Zhang and Pan, 2021	CDCIWG	20	20	NA	NA	61.0 ± 6.88	60.28 ± 5.49	10.75 ± 2.39	10.45 ± 2.4	25.05 ± 1.59	25.23 ± 1.63	22.64 ± 1.81	22.58 ± 1.56	rTMS	Sham rTMS	Moca, MMSE
Wen et al., 2018	Peterson	23	22	60.9	45.5	64.17 ± 5.21	65.91 ± 4.93	NA	NA	NA	NA	22.30 ± 1.36	21.82 ± 1.62	rTMS	Sham rTMS	Moca, RBMT
Deng et al., 2019	Peterson	25	25	60	56	67.2 ± 5.89	68.3 ± 5.6	NA	NA	NA	NA	22.68 ± 1.91	23.80 ± 1.85	rTMS	Sham rTMS	Moca
Quan et al., 2025	DSM-5 and Peterson	33	32	57.6	65.6	65.73 ± 3.19	66.81 ± 3.19	9.58 ± 3.46	9.5 ± 3.18	22.97 ± 1.78	23.13 ± 1.64	21.88 ± 1.75	21.53 ± 1.69	rTMS	Sham rTMS	Moca, MMSE
Zhou and Chen, 2023	NA	30	30	60	56.7	73.42 ± 4.53	73.47 ± 4.59	NA	NA	24.06 ± 2.18	23.72 ± 2.22	22.94 ± 1.96	23.49 ± 1.36	rTMS + Compound sea snake capsule	Compound sea snake capsule	Moca, MMSE, RBMT
Sun and Ma, 2015	NA	40	40	57.5	50	65.4 ± 5.6	63.4 ± 8.2	NA	NA	NA	NA	21.64 ± 2.16	20.09 ± 2.34	rTMS + Cognitive training	Cognitive training	Moca
Liu et al., 2025	Peterson	55	55	32.7	43.6	67.8 ± 4.5	68.0 ± 4.6	11.0 ± 2.6	11.2 ± 2.7	NA	NA	23.2 ± 1.8	22.6 ± 2.0	rTMS + Tai Chi Chuan	Sham rTMS + Tai Chi Chuan	Moca
Lv et al., 2024	Peterson	39	21	46.2	57.1	66.74 ± 1.08	64.95 ± 8.72	11.95 ± 2.69	11.76 ± 3.55	26.69 ± 3.11	26.95 ± 3.42	22.46 ± 3.61	23.43 ± 4.98	rTMS	Sham rTMS	Moca
Chen et al., 2025	Peterson	17	10	29.4	30	67.0 ± 7.0	68.00 ± 8.75	12 ± 1.5	12 ± 6.25	28.00 ± 2.00	27.00 ± 1.50	24.00 ± 7.00	24.5 ± 4.25	rTMS	Sham rTMS	Moca
Gy et al., 2021	DSM-5 and NIA-AA	12	12	58.3	25	66.1 ± 5.5	67.2 ± 4.8	15.2 ± 4.8	14.7 ± 4.1	28.6 ± 1.5	28.5 ± 1.1	23.2 ± 1.5	22.2 ± 1.5	rTMS	Sham rTMS	Moca, MMSE
Gy et al., 2021	DSM-5 and NIA-AA	11	11	27.3	54.5	NA	NA	NA	NA	28.4 ± 1.4	29.2 ± 0.9	23 ± 1.5	26.2 ± 1.6	rTMS	Sham rTMS	Moca, MMSE
Riccitelli et al., 2025	Peterson	12	12	58.3	41.7	75.6 ± 4.1	74.3 ± 5.3	11.6 ± 2.7	12.6 ± 3.3	NA	NA	21.1 ± 2.6	20.7 ± 3.5	rTMS	Sham rTMS	Moca
Drumond Marra et al., 2015	Peterson	15	19	40	31.6	65.1 ± 3.5	65.2 ± 4.1	15.1 ± 4.4	12.4 ± 4.7	NA	NA	24.5 ± 1.8	24.2 ± 2.3	rTMS	Sham rTMS	RBMT

Data are presented as mean ± standard deviation (SD) or percentage, as appropriate. n1 represents the intervention group and n2 represents the control group. NA indicates that the corresponding information was not reported in the original study. rTMS, repetitive transcranial magnetic stimulation; MoCA, Montreal Cognitive Assessment; MMSE, Mini-Mental State Examination; RBMT, Rivermead Behavioral Memory Test; DSM-5, Diagnostic and Statistical Manual of Mental Disorders, Fifth Edition; NIA-AA, National Institute on Aging–Alzheimer’s Association; CDCIWG: Chinese Dementia and Cognitive Impairment Working Group, 2018; NA, not available.

**TABLE 2:** rTMS stimulation characteristics of the included studies.

Study	Frequency (Hz)	Intensity	Coil type	Pulses per session	Treatment duration	Total sessions	Stimulation site	Target-localization method
Zhu et al., 2020	10	80%	Figure-eight coil	400	4 weeks	20	Left DLPFC	5 cm method
Han et al., 2013	20	80%	Figure-eight coil	600	8 weeks	40	B-DLPFC	Scalp landmark-based localization
Zhang and Pan, 2021	10	80%	Figure-eight coil	2500	8 weeks	40	Left DLPFC	Scalp landmark-based localization
Wen et al., 2018	10	80%	Figure-eight coil	400	4 weeks	20	Left DLPFC	Scalp landmark-based localization
Deng et al., 2019	15	80%	Figure-eight coil	900	12 weeks	15	Left DLPFC	Scalp landmark-based localization
Quan et al., 2025	10	80%	Figure-eight coil	400	4 weeks	20	Left DLPFC	International 10 - 20 EEG system
Zhou and Chen, 2023	10	80%	Figure-eight coil	1200	12 weeks	36	B-DLPFC	Scalp landmark-based localization
Sun and Ma, 2015	15	80%–110%	Figure-eight coil	7200	8 weeks	48	Left DLPFC/PFC	International 10–20 EEG system
Liu et al., 2025	1	80%	Figure-eight coil	1200	6 weeks	30	Right DLPFC	Neuronavigation system
Lv et al., 2024	20	100%	Figure-eight coil	1600	4 weeks	20	Left angular gyrus	MNI coordinates
Chen et al., 2025	20	100%	Figure-eight coil	1600	4 weeks	20	Left angular gyrus	MNI coordinates
Gy et al., 2021	5	100%	Figure-eight coil	1500	10 weeks	30	Left DLPFC	Beam-F3 position
Gy et al., 2021	5	100%	Figure-eight coil	1500	10 weeks	30	Left DLPFC	Beam-F3 position
Riccitelli et al., 2025	20	100%	Figure-eight coil	2000	2 weeks	10	rTPJ + mPFC	MNI coordinates
Drumond Marra et al., 2015	10	110%	Figure-eight coil	2000	10 consecutive weekdays	10	Left DLPFC	5 cm method

Stimulation intensity is expressed as a percentage of the resting motor threshold (RMT). Pulses per session refers to the total number of magnetic pulses delivered during each treatment session. rTMS, repetitive transcranial magnetic stimulation; DLPFC, dorsolateral prefrontal cortex; B-DLPFC, bilateral dorsolateral prefrontal cortex; PFC, prefrontal cortex; rTPJ, right temporoparietal junction; mPFC, medial prefrontal cortex; MNI, Montreal Neurological Institute; EEG, electroencephalography.

All studies used figure-of-eight coils. Stimulation frequency ranged from 1 to 20 Hz, with the majority applying high-frequency protocols (≥ 5 Hz), and stimulation intensity ranged from 80 to 110% of resting motor threshold. The left DLPFC was the most common target, followed by the bilateral or right DLPFC and other regions. Treatment duration, number of sessions, and total pulse exposure varied substantially across studies. Most studies (11/14) applied rTMS as a stand-alone intervention, whereas a minority combined it with adjunctive therapies.

### 3.2 Effects of rTMS on cognitive performance

Fourteen datasets (*n* = 672; rTMS *n* = 352, sham *n* = 320) reported post-intervention MoCA scores (Figure 2A). Random-effects pooling showed higher post-intervention MoCA scores in the rTMS group than in the sham group (Hedges’ *g* = 1.46, 95% CI 0.97 to 1.94; *p* < 0.001). However, substantial heterogeneity was observed (I^2^ = 86.0%, 95% CI 57.5% to 93.2%). Most point estimates favored active rTMS; however, the pooled estimate should be interpreted as an average across heterogeneous studies rather than as a precise estimate of the expected treatment effect. Five datasets contributed to the MMSE analysis (Figure 2B). rTMS was associated with a significant improvement compared with sham (*g* = 1.08, 95% CI 0.61 to 1.54; *p* < 0.001), with moderate heterogeneity (I^2^ = 57.6%, 95% CI 0.0% to 85.0%). Three datasets assessed RBMT outcomes (Figure 2C), showing a significant benefit of rTMS (*g* = 0.99, 95% CI 0.24 to 1.75; *p* = 0.010), despite substantial heterogeneity (I^2^ = 76.9%, 95% CI 0.0% to 93.7%).

**FIGURE 2:**
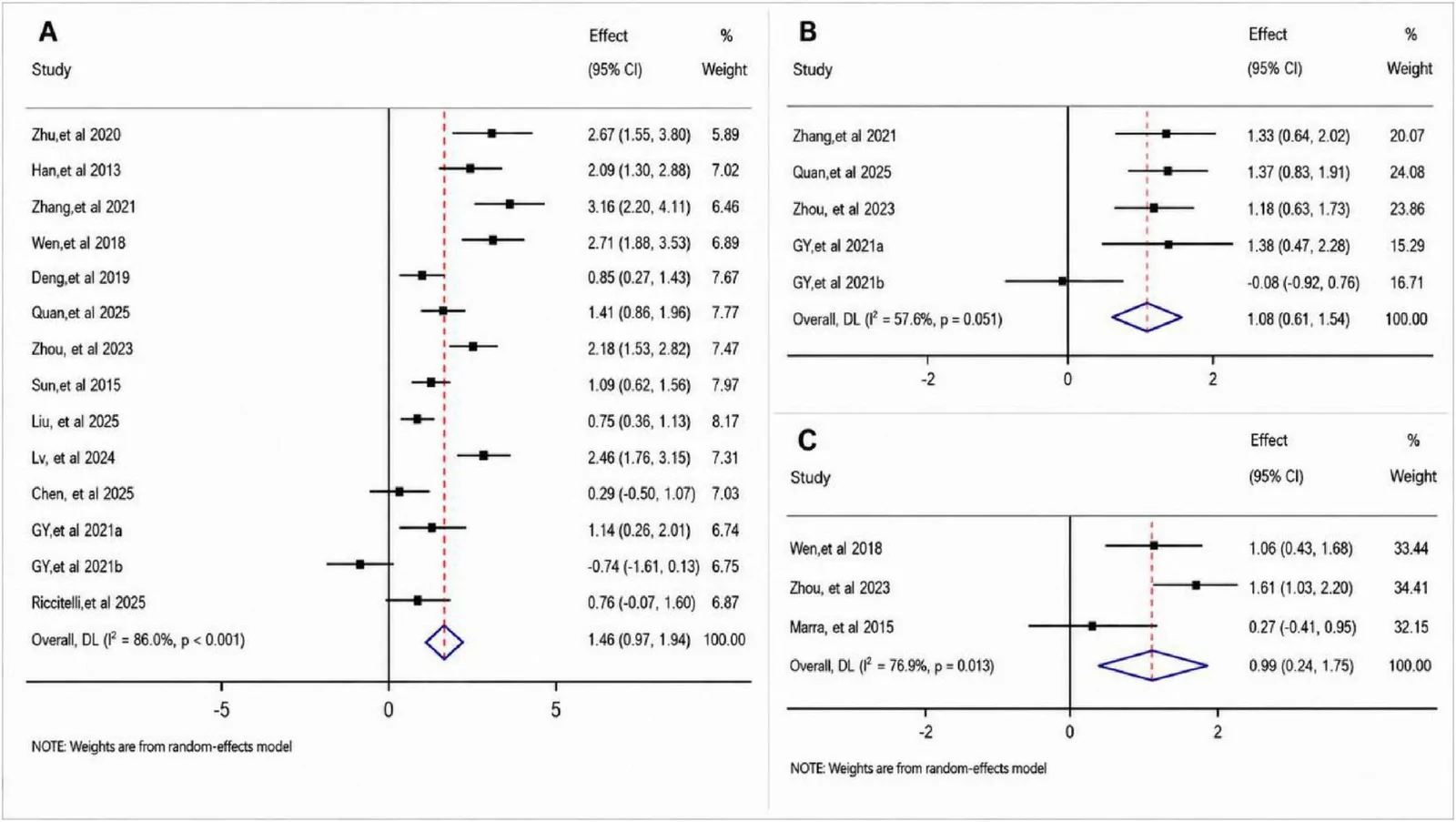
Forest plots of the effects of rTMS on cognitive outcomes in patients with mild cognitive impairment. **(A)** Montreal Cognitive Assessment (MoCA; 14 datasets); **(B)** Mini-Mental State Examination (MMSE; 5 datasets); and **(C)** Rivermead Behavioral Memory Test (RBMT; 3 datasets). Effect sizes are presented as Hedges’ g with 95% confidence intervals (CIs) and were pooled using random-effects models. Positive effect sizes indicate better post-intervention cognitive performance in the rTMS group relative to the sham or control group. Squares represent individual dataset effect estimates, with square size proportional to study weight; horizontal lines indicate 95% CIs, and diamonds represent pooled effect estimates. The solid vertical line indicates the null effect (Hedges’ *g* = 0), and the dashed vertical line indicates the pooled effect estimate for each outcome. Between-study heterogeneity is reported using the I^2^ statistic.

### 3.3 Subgroup analyses

Subgroup analyses were performed to explore potential sources of heterogeneity in MoCA outcomes (Supplementary Figures 1–7). According to risk of bias, the pooled effect was numerically smaller in the low-risk subgroup than in the high/unclear-risk subgroup; however, the between-subgroup difference was not statistically significant (*p* = 0.114) (Supplementary Figure 1). Notably, the low-risk subgroup comprised only two datasets derived from a single crossover trial. No significant subgroup differences were observed according to adjunctive interventions (*p* = 0.681) or diagnostic criteria (*p* = 0.935) (Supplementary Figures 2, 3, respectively). Among stimulation parameters, a significant subgroup difference was observed for stimulation frequency (*p* = 0.021), with high-frequency stimulation associated with a larger pooled effect than low-frequency stimulation (Supplementary Figure 4). In contrast, the between-subgroup differences according to stimulation intensity (*p* = 0.099) and cumulative pulse exposure (*p* = 0.143) were not statistically significant (Supplementary Figures 5, 6). A significant subgroup difference was also observed according to stimulation site (*p* < 0.001), with bilateral DLPFC stimulation showing the largest pooled effect, followed by left DLPFC stimulation, whereas right DLPFC and other targets showed smaller or less consistent effects (Supplementary Figure 7).

### 3.4 Meta-regression analysis

Exploratory univariable meta-regression analyses did not identify significant associations between the MoCA effect size and mean age (β = −0.105, 95% CI −0.105, 95% CI d *m* = 0.171), baseline MoCA score (β = −0.045, 95% CI −0.710 to 0.621; *p* = 0.884), number of treatment sessions (β = −0.005, 95% CI −0.069 to 0.058; *p* = 0.856), treatment duration (β = 0.028, 95% CI −0.214 to 0.271; *p* = 0.800), or cumulative pulse exposure per 10,000 pulses (β = −0.019, 95% CI −0.085 to 0.047; *p* = 0.539). Substantial residual heterogeneity remained across all models (residual I^2^ = 84.96%–85.86%), indicating that these continuous study-level moderators did not adequately explain the observed between-study variability.

### 3.5 Sensitivity analysis of MoCA

Sensitivity analyses were performed to assess the robustness of the primary MoCA findings. In the leave-one-out analysis, sequential omission of each dataset yielded consistently positive pooled effect estimates, with Hedges’ g values remaining within a relatively narrow range of approximately 1.24–1.46. All corresponding 95% confidence intervals excluded zero (Supplementary Figure 8). No individual dataset materially altered the direction or statistical significance of the pooled association, indicating that the primary MoCA finding was not driven by any single dataset. To specifically assess the influence of the crossover trial, a separate sensitivity analysis excluding both comparisons from Gy et al. (2021) yielded a pooled effect of Hedges’ *g* = 1.65 (95% CI 1.16 to 2.13; *p* < 0.001), with substantial heterogeneity remaining (I^2^ = 84.5%, 95% CI 47.2% to 92.7%) (Supplementary Figure 9). Thus, exclusion of the crossover trial did not materially alter the direction or statistical significance of the primary association. Results were also consistent across fixed-effect and random-effects models. Influence diagnostics identified several datasets contributing to heterogeneity, although their exclusion did not materially alter the direction of the pooled association.

### 3.6 Publication bias

Publication bias was assessed for the MoCA outcome using 12 independent studies after exclusion of the crossover study by Gy et al. (2021). For the MMSE and RBMT outcomes, funnel plots were provided for descriptive purposes only because of the limited number of studies; no formal statistical tests for small-study effects were performed. For the MoCA outcome, visual inspection of the funnel plot suggested some asymmetry (Supplementary Figure 10), and Egger’s regression test indicated significant small-study effects (bias = 5.39, SE = 2.07, 95% CI 0.78 to 10.00; *p* = 0.026). A trim-and-fill sensitivity analysis (Supplementary Figure 11) imputed no potentially missing studies, and the pooled effect estimate remained unchanged (Hedges’ *g* = 1.69, 95% CI 1.15 to 2.22). The MMSE and RBMT funnel plots are shown in Supplementary Figures 12, 13 and are interpreted descriptively only.

### 3.7 Risk-of-bias assessment in individual studies

Risk-of-bias assessments are summarized in Supplementary Table 2. Across the 14 unique trials, random sequence generation was rated as low risk in 8 trials and unclear risk in 6, whereas allocation concealment was rated as low risk in 2 and unclear risk in 12. Blinding of participants and personnel represented the main methodological concern, with 5 trials rated as low risk, 2 as unclear risk, and 7 as high risk. Incomplete outcome data were rated as low risk in 13 trials and unclear risk in 1, while selective reporting was rated as low risk in 13 trials and high risk in 1. Other bias was rated as low risk in 5 trials and unclear risk in 9. Overall, 2 trials were classified as low risk of bias, 4 as unclear risk, and 8 as high risk. Two trials, Gy et al. (2021) and Drumond Marra et al. (2015), were judged to be at low risk of bias across all domains. Study-level risk-of-bias classifications are summarized in Supplementary Table 2.

### 3.8 Adverse events

Ten of the 15 included datasets (67%) reported quantitative adverse-event data, while the remaining five provided no usable safety information. Among datasets with available data, adverse events occurred in 29/243 participants (11.9%) in the rTMS group and 21/231 (9.1%) in the sham group. The most commonly reported events were transient headache and dizziness. No serious adverse events attributable to rTMS were reported. Pooled analysis showed no significant difference in adverse-event risk between groups (RR = 1.25, 95% CI 0.76 to 2.04; *p* = 0.374).

## 4 Discussion

This meta-analysis of randomized controlled trials found that rTMS was associated with higher post-intervention scores on measures of global cognitive function and memory in patients with MCI. Although the direction of effect generally favored rTMS across the MoCA, MMSE, and RBMT outcomes, the large pooled effect for MoCA was accompanied by substantial heterogeneity, indicating considerable uncertainty in the magnitude and generalizability of the estimated benefit. This overall direction of the finding is consistent with previous studies (Jiang et al., 2020; Zhang et al., 2021; Wang et al., 2025). However, an earlier systematic review reported more modest improvements in MoCA scores, with a standardized mean difference of approximately 0.3–0.5 (Chou et al., 2020), further supporting cautious interpretation. This caution is reinforced by the overall risk-of-bias profile: only 2 of the 14 trials were classified as having a low overall risk of bias, whereas 8 were rated as high risk and 4 as unclear, with blinding representing a particular methodological concern. Although the between-subgroup difference according to risk of bias was not statistically significant (*p* = 0.114), the numerically larger pooled effect among high/unclear-risk trials and the limited amount of low-risk evidence further support cautious interpretation of the relatively large pooled MoCA effect. Moreover, statistically significant improvements in cognitive test performance should not be assumed to represent clinically meaningful functional benefits, because the included studies did not consistently assess clinically anchored response thresholds or patient-centered functional outcomes.

The clinical interpretation of these findings also depends on the characteristics of the cognitive measures used. The MoCA, MMSE, and RBMT are commonly used tools in the field of cognitive disorders. The MMSE demonstrates limited sensitivity for early MCI due to a ceiling effect, whereas the MoCA shows greater sensitivity to deficits in executive function and visuospatial abilities (Islam et al., 2023). The RBMT focuses on episodic memory and aligns more closely with everyday memory functioning, but it may overlook impairments in other cognitive domains, such as executive function, attention (Fonte et al., 2019). In addition, domain-specific outcomes involving executive function, attention, language, and visuospatial abilities were inconsistently reported and assessed using heterogeneous instruments across studies, precluding reliable domain-specific meta-analyses. Therefore, although statistically significant pooled effects were observed across the available cognitive measures, it remains uncertain whether these changes translate into meaningful improvements in daily functioning, independence, or quality of life. Future trials should combine standardized cognitive measures with broader neuropsychological assessments and clinically meaningful functional outcomes to better establish the clinical relevance of rTMS in MCI.

Substantial heterogeneity remained a central feature of the present analysis. Previous studies have identified several potential sources of variability in rTMS trials, including differences in stimulation frequency (high-frequency vs. low-frequency) (Bai et al., 2022; Cui et al., 2022), intensity, stimulation targets such as the DLPFC, parietal-hippocampal regions (Wei et al., 2022; Chen H. F. et al., 2023; Lv et al., 2024), and coil type (Lee et al., 2020; Zhang C. et al., 2025). In the present subgroup analyses, statistically significant between-subgroup differences were observed for stimulation frequency (*p* = 0.021) and stimulation site (*p* < 0.001), whereas differences according to risk of bias (p = 0.114), adjunctive interventions (*p* = 0.681), diagnostic criteria (*p* = 0.935), stimulation intensity (*p* = 0.099), and cumulative pulse exposure (*p* = 0.143) were not statistically significant. These study-level comparisons should nevertheless be interpreted as exploratory rather than as evidence of causal or definitive effect modification, particularly because several stimulation and study characteristics were interrelated and some subgroups contained relatively few studies. Exploratory meta-regression analyses likewise did not identify significant associations between the MoCA effect size and mean age, baseline MoCA score, number of treatment sessions, cumulative pulse exposure, or treatment duration. However, substantial residual heterogeneity remained, suggesting that the available study-level characteristics did not adequately account for the observed between-study variability. Given the limited number of independent studies, these null meta-regression findings should not be interpreted as evidence that these factors are unrelated to treatment response. Thus, the pooled effect should be regarded as an average across clinically and methodologically heterogeneous rTMS protocols rather than as the expected effect of a single standardized intervention.

The risk-of-bias subgroup analysis showed a numerically larger pooled effect among high/unclear-risk trials than among low-risk trials, but the between-subgroup difference was not statistically significant (*p* = 0.114). Moreover, the low-risk subgroup comprised only two datasets derived from a single crossover trial, substantially limiting the precision of this comparison. Accordingly, the present data do not establish study quality as a definitive effect modifier. Nevertheless, methodological quality remains important when interpreting the relatively large pooled effect. Blinding represents a particular challenge in non-invasive brain stimulation studies (Gogulski et al., 2023), because even when sham procedures such as coil tilting or shielding pads are used, participants may distinguish treatment allocation through differences in scalp sensations, auditory cues, or coil positioning. Such inadvertent unblinding could increase expectancy or placebo-related effects and potentially inflate estimated treatment effects. The overall pooled estimate should therefore continue to be interpreted cautiously, although the present subgroup analysis does not provide statistical evidence that risk of bias significantly modified the treatment effect.

In contrast to the findings for frequency and stimulation site, the between-subgroup differences according to stimulation intensity (*p* = 0.099) and cumulative pulse exposure (*p* = 0.143) were not statistically significant. Therefore, the present results do not support a clear dose-response relationship or an optimal intensity or pulse dose. Although stimulation intensity and cumulative pulse exposure may influence neuroplastic responses under some experimental conditions (Halawa et al., 2021; Chen S. et al., 2023), higher stimulation doses do not necessarily translate into greater clinical benefit. Moreover, treatment-related variables are often correlated, making it difficult to isolate the independent contribution of intensity or pulse exposure in study-level subgroup analyses. Direct comparative trials that systematically vary individual stimulation parameters are needed before conclusions regarding dose optimization can be made.

Regarding stimulation site, the bilateral dorsolateral prefrontal cortex (DLPFC) stimulation was associated with a larger pooled effect in the subgroup analysis. However, this finding should not be interpreted as evidence that bilateral DLPFC stimulation is superior, given the small number of studies in some subgroups and potential confounding by study quality and other protocol characteristics. The DLPFC nevertheless remains a biologically plausible target because of its role in cognitive control, working memory, and executive function, as well as its functional connections with memory-related brain regions such as the hippocampus (Balderston et al., 2020; Wu et al., 2022; Zhao et al., 2025). Stimulation of the DLPFC may therefore influence cognition not only through local modulation of cortical excitability but also through distributed network-level effects (Ahmed et al., 2012; Turriziani et al., 2019; Esposito et al., 2022). More broadly, rTMS is one of several non-invasive brain-stimulation approaches investigated for cognitive enhancement. Studies in healthy adults have suggested that prefrontal, particularly DLPFC-targeted, rTMS can modulate attention, working memory, executive function, and other cognitive processes, although the magnitude and direction of these effects vary according to stimulation protocol, cognitive task, and baseline brain state (Ebrahimzadeh et al., 2025). In addition, differences in total sessions and outcome assessment time points across studies are important factors contributing to heterogeneity (Asgarinejad et al., 2024). Regarding concomitant medication, some studies suggest that combining rTMS with neurotransmitter-targeting drugs, including donepezil, may influence cognitive and network-level outcomes (Li et al., 2023; Shou et al., 2024), but most studies did not control for this variable. These protocol and clinical factors further complicate the interpretation of target-specific effects.

Several complementary biological mechanisms may contribute to the cognitive effects of rTMS. At the synaptic level, repeated stimulation may induce long-term potentiation- or long-term depression-like plasticity and influence metaplasticity, thereby altering the responsiveness of cortical circuits to subsequent stimulation (Hoogendam et al., 2010). Activity-dependent modulation of neurotrophic factors, particularly brain-derived neurotrophic factor (BDNF), may further support synaptic remodeling and plasticity (Müller et al., 2000). rTMS may also modify glutamatergic and GABAergic neurotransmission and influence dopaminergic signaling relevant to attention, learning, and executive control (Strafella et al., 2001; Godfrey et al., 2021). At the systems level, prefrontal stimulation may propagate beyond the stimulated cortex and reorganize connectivity within distributed cognitive networks, including the frontoparietal and default mode networks, both of which are implicated in cognitive dysfunction in MCI (Esposito et al., 2022). rTMS may additionally alter regional cerebral blood flow and metabolic activity, potentially modifying metabolic support within cognition-related networks (Kinney and Hanlon, 2022). These mechanisms are likely to interact across synaptic, neurochemical, network, and vascular levels; however, their specific contributions to the cognitive effects observed in MCI remain to be established.

The present findings can also be viewed in the broader context of rTMS applications in major depressive disorder (MDD), where DLPFC-targeted rTMS has an established therapeutic role and cognitive improvement is generally considered a secondary outcome. Although MCI and MDD differ in their primary clinical targets, both may involve modulation of prefrontal networks and neuroplasticity. Research in depression has also explored individualized prediction of rTMS response using time-frequency EEG, non-linear signal processing, reinforcement-learning approaches, and machine-learning models (Hasanzadeh et al., 2019; Ebrahimzadeh et al., 2021, 2023, 2024; Nikravan and Ebrahimzadeh, 2021). These approaches may provide a useful methodological framework for future MCI studies integrating neurophysiological, cognitive, and clinical features to improve patient selection. However, protocols and predictive models developed in MDD cannot be directly extrapolated to MCI, for which the current evidence remains limited and heterogeneous.

No statistically significant between-subgroup differences were observed according to diagnostic criteria or adjunctive interventions. However, these null subgroup tests should not be interpreted as evidence of equivalence across diagnostic frameworks or treatment contexts. Diagnostic criteria varied across the included studies, and many studies either did not clearly differentiate etiological subtypes or lacked sufficient statistical power for such comparisons. Furthermore, concomitant medication use was incompletely and inconsistently reported, precluding reliable assessment of whether background pharmacotherapy modified the effects of rTMS. Medication status may therefore represent an additional source of between-study heterogeneity. Therefore, the absence of significant subgroup differences should not be interpreted as evidence of no effect modification, and future trials should improve diagnostic stratification and reporting of concomitant medications and baseline characteristics.

Sensitivity analyses showed that the main findings were generally robust. The overall effect size remained statistically significant after the sequential exclusion of individual studies, and the results were consistent across different statistical models. These findings indicate that the observed association between rTMS and cognitive improvement was not driven by any single study or by the choice of statistical model, thereby supporting the stability of the overall direction of effect. Importantly, exclusion of both comparisons from the crossover trial by Gy et al. (2021) yielded a pooled effect of Hedges’ *g* = 1.65 (95% CI 1.16 to 2.13; *p* < 0.001), with I^2^ = 84.5%. Thus, inclusion of the crossover trial did not materially alter the direction or statistical significance of the overall association. Nevertheless, treating the two treatment periods as independent observations does not account for their within-subject correlation and may underestimate the variance of the pooled estimate.

Potential small-study effects provide an additional reason for caution. Egger’s regression test indicated significant small-study effects for the MoCA outcome, although the trim-and-fill analysis imputed no missing studies and did not materially change the pooled estimate. Given the substantial between-study heterogeneity, funnel-plot asymmetry cannot be attributed solely to publication bias and may also reflect differences in sample size, study quality, stimulation protocols, or other study characteristics. For the MMSE and RBMT outcomes, the small numbers of available studies precluded reliable statistical assessment of funnel-plot asymmetry, and the corresponding funnel plots should therefore be interpreted descriptively only.

Among the datasets reporting quantitative safety outcomes, no statistically significant increase in reported adverse events was observed with rTMS compared with sham stimulation. Reported adverse events were predominantly mild and transient, and no serious adverse events attributable to rTMS were reported. However, quantitative adverse-event data were available for only 10 of the 15 datasets, limiting the completeness of the safety evidence. In particular, the available data primarily reflect short-term adverse events and do not permit firm conclusions regarding long-term tolerability or rare adverse events. This limitation is clinically relevant in MCI, which predominantly affects older adults who may have greater medical comorbidity, polypharmacy, and vulnerability to treatment-related adverse effects. Therefore, the absence of a statistically significant increase in reported adverse events should not be interpreted as definitive evidence of safety in routine clinical practice. Previous studies have also often reported tolerability without systematically documenting the incidence, severity, causal relationship to treatment, and outcomes of adverse events (Rossi et al., 2021). More comprehensive and standardized safety reporting is therefore needed to characterize the long-term safety profile of rTMS in this population.

From a clinical perspective, the available evidence suggests that rTMS may improve cognitive test performance in MCI, but it does not yet permit reliable identification of patients most likely to benefit or establish an optimal stimulation protocol. Although significant between-subgroup differences were observed for stimulation frequency and site, the findings remain exploratory and should not be interpreted as demonstrating that high-frequency or bilateral DLPFC stimulation is superior. Evidence is also insufficient to define the optimal stimulation intensity, cumulative pulse exposure, treatment duration, expected time course or durability of benefit, or the need for maintenance sessions. Future trials should directly compare prespecified stimulation protocols and evaluate longer-term outcomes, maintenance strategies, and integration with pharmacological or cognitive interventions (Ebrahimzadeh et al., 2023).

However, several limitations should be considered. First, the limited number of trials within several subgroups reduced statistical power and the precision of subgroup estimates. Second, domain-specific cognitive outcomes were inconsistently reported, preventing quantitative assessment of whether rTMS preferentially affects particular cognitive domains. Third, substantial residual heterogeneity remained after the subgroup and meta-regression analyses, limiting the precision and generalizability of the overall pooled effect. Although exploratory meta-regression analyses examined mean age, baseline MoCA score, number of treatment sessions, cumulative pulse exposure, and treatment duration, none adequately explained the observed heterogeneity. Other potentially relevant sources of variability, including target-localization method, sham methodology, and outcome assessment timing, could not be formally evaluated because of limited and inconsistent reporting. In addition, correlations among study and stimulation characteristics made it difficult to isolate the independent contribution of any single factor. The subgroup and meta-regression findings should therefore be regarded as exploratory rather than as evidence of definitive effect modifiers. Because multiple exploratory subgroup comparisons were performed without adjustment for multiplicity and some subgroups contained very few studies, nominally significant subgroup findings should be interpreted cautiously. Fourth, because the primary analyses were based on post-intervention scores, residual baseline imbalances in small trials may have influenced individual effect estimates despite randomization. Fifth, due to the absence of within-subject correlation data, one crossover trial was analyzed as two independent comparisons, a methodological approach that may have contributed to imprecision in the pooled estimates. However, exclusion of both comparisons from this crossover trial yielded a similar overall conclusion, suggesting that inclusion of this study did not materially affect the direction or statistical significance of the primary finding. Finally, although potential small-study effects were formally assessed for the primary MoCA outcome, the small numbers of studies contributing to the MMSE and RBMT analyses precluded reliable statistical assessment of funnel-plot asymmetry. The funnel plots for these secondary outcomes should therefore be regarded as descriptive only, and publication bias or small-study effects cannot be excluded.

## 5 Conclusion

Overall, the available randomized evidence suggests that rTMS may improve cognitive test performance in patients with MCI. However, substantial between-study heterogeneity limits the precision and generalizability of the pooled effect. Exploratory subgroup analyses suggested variation according to stimulation frequency and site, but these findings do not establish definitive effect modifiers or optimal stimulation parameters. Other examined subgroup factors, including risk of bias, did not show statistically significant between-subgroup differences. No significant increase in reported short-term adverse events was observed, although safety reporting was incomplete. Well-designed, adequately powered trials with rigorous blinding, standardized stimulation protocols, clinically meaningful outcome measures, and longer follow-up are needed to clarify the magnitude, durability, and clinical relevance of rTMS effects in MCI.
