# Supplementary material for: Effects and safety of repetitive transcranial magnetic stimulation on cognitive outcomes in mild cognitive impairment: a systematic review and meta-analysis
Source: Front Aging Neurosci. 2026 Sep 24;18:1894724. doi: 10.3389/fnagi.2026.1894724 (PMC13646044; doi:10.3389/fnagi.2026.1894724)
Supplement: Supplementary file 1 [file Table_1.docx]

**Supplementary Table S1. Exploratory univariable meta-regression of study-level moderators for the MoCA outcome**

| **Moderator** | **β** | **SE** | **95% CI** | **P value** | **τ²** | **Residual I²** | **Adj. R²** |
| --- | --- | --- | --- | --- | --- | --- | --- |
| Mean age (per year) | -0.105 | 0.071 | -0.264 to 0.054 | 0.171 | 0.6581 | 84.96% | 8.17% |
| Baseline MoCA (per point) | -0.045 | 0.299 | -0.710 to 0.621 | 0.884 | 0.8071 | 85.86% | -12.63% |
| Treatment sessions (per session) | -0.005 | 0.028 | -0.069 to 0.058 | 0.856 | 0.8080 | 85.61% | -12.75% |
| Treatment duration (per week) | 0.028 | 0.109 | -0.214 to 0.271 | 0.800 | 0.8025 | 85.80% | -11.99% |
| Cumulative pulses (per 10,000) | -0.019 | 0.030 | -0.085 to 0.047 | 0.539 | 0.7762 | 85.42% | -8.32% |

Note. All models included 12 studies and were fitted separately. β represents the change in Hedges' g per one-unit increase in the moderator. For cumulative pulse exposure, one unit corresponds to 10,000 pulses. Negative adjusted R² values should not be interpreted as 'negative explained heterogeneity'; they indicate that the moderator did not improve explanation of between-study variance relative to the intercept-only model.

**Supplementary Figure S1. Subgroup analysis of the MoCA outcome according to study-level risk of bias.**

Forest plot illustrating the effects of rTMS on MoCA scores stratified by study-level risk of bias. Studies classified as low risk and high/unclear risk were analyzed separately. Pooled estimates are presented as Hedges’ g with 95% confidence intervals using a random-effects model. The test for subgroup differences was performed to evaluate whether methodological quality was associated with variation in treatment effects.

**Supplementary Figure S2. Subgroup analysis of the MoCA outcome according to treatment.**

Studies were stratified according to treatment modality, with subgroup 1 representing rTMS alone and subgroup 2 representing rTMS combined with other interventions. Pooled effect estimates are presented as Hedges’ g with 95% confidence intervals using a random-effects model. The between-subgroup difference was not statistically significant, indicating no evidence of a differential treatment effect according to the use of adjunctive interventions.

**Supplementary Figure S3. Subgroup analysis of the MoCA outcome according to diagnostic criteria.**

Studies were stratified according to the diagnostic criteria used for MCI, with subgroup 1 representing Petersen criteria, subgroup 2 representing non-Petersen criteria, and subgroup 3 representing unclear diagnostic criteria. Pooled effect estimates are presented as Hedges’ g with 95% confidence intervals using a random-effects model. The between-subgroup difference was not statistically significant (p = 0.935), providing no evidence that diagnostic criteria significantly modified the pooled effect of rTMS on MoCA scores.

**Supplementary Figure S4. Subgroup analysis of the MoCA outcome according to stimulation frequency**

Studies were stratified according to stimulation frequency, with subgroup 1 representing low-frequency stimulation (1 Hz) and subgroup 2 representing high-frequency stimulation (≥5 Hz). Pooled effect estimates are presented as Hedges’ g with 95% confidence intervals using a random-effects model. A statistically significant between-subgroup difference was observed (p = 0.021), with high-frequency stimulation showing a larger pooled effect than low-frequency stimulation. Given the limited number of studies in the low-frequency subgroup, this finding should be interpreted as exploratory.

**Supplementary Figure S5. Subgroup analysis of the MoCA outcome according to stimulation intensity**

Studies were stratified according to stimulation intensity, with subgroup 1 representing stimulation at <100% of resting motor threshold (RMT) and subgroup 2 representing stimulation at ≥100% RMT. Pooled effect estimates are presented as Hedges’ g with 95% confidence intervals using a random-effects model. The between-subgroup difference was not statistically significant (p = 0.099), providing no evidence that stimulation intensity significantly modified the pooled effect of rTMS on MoCA scores.

**Supplementary Figure S6. Subgroup analysis of the MoCA outcome according to cumulative pulse exposure**

Studies were stratified according to total pulse exposure, with subgroup 1 representing <30,000 pulses and subgroup 2 representing ≥30,000 pulses. Pooled effect estimates are presented as Hedges’ g with 95% confidence intervals using a random-effects model. The between-subgroup difference was not statistically significant (p = 0.143), providing no evidence that cumulative pulse exposure significantly modified the pooled effect of rTMS on MoCA scores.

**Supplementary Figure S7. Subgroup analysis of the MoCA outcome according to stimulation site.**

Studies were stratified according to stimulation site, with subgroup 1 representing left dorsolateral prefrontal cortex (DLPFC), subgroup 2 representing bilateral DLPFC, subgroup 3 representing right DLPFC, and subgroup 4 representing other stimulation targets. Pooled effect estimates are presented as Hedges’ g with 95% confidence intervals using a random-effects model. A statistically significant between-subgroup difference was observed (p < 0.001), with bilateral DLPFC stimulation showing the largest pooled effect, followed by left DLPFC stimulation, whereas right DLPFC and other stimulation targets showed smaller or less consistent pooled effects. Given the limited number of studies within some subgroups, these findings should be interpreted as exploratory and do not establish the superiority of any specific stimulation site.

**Supplementary Figure S8. Leave-one-out sensitivity analysis of the MoCA outcome.**

This plot shows the pooled effect estimate recalculated after sequential omission of each individual dataset. Circles indicate the pooled Hedges’ g after omission of the named study, and horizontal lines represent the corresponding 95% confidence intervals. The results show that no single dataset materially altered the direction or statistical significance of the overall pooled association, supporting the robustness of the primary MoCA findings.

**Supplementary Figure S9. Sensitivity analysis of the MoCA outcome excluding the crossover trial.**

The sensitivity analysis was performed after excluding both comparisons from the crossover trial by Gy et al. (2021) to account for the potential dependence between repeated observations from the same participants. Pooled effect estimates are presented as Hedges’ g with 95% confidence intervals using a random-effects model. After exclusion of the crossover trial, rTMS remained associated with higher post-intervention MoCA scores compared with sham or control interventions (Hedges’ g = 1.65, 95% CI 1.16 to 2.13; p < 0.001), although substantial between-study heterogeneity persisted (I² = 84.5%). The direction and statistical significance of the pooled association were therefore not materially altered by exclusion of the crossover trial.

**Supplementary Figure S10. Funnel plot for the MoCA outcome.**

The funnel plot was constructed using independent studies contributing to the MoCA analysis; the crossover trial by Gy et al. (2021) was excluded to avoid dependence between its two comparisons. Visual inspection suggested some funnel-plot asymmetry. Egger’s regression test indicated significant small-study effects (bias = 5.39, SE = 2.07, 95% CI 0.78 to 10.00; p = 0.026). Given the substantial between-study heterogeneity, the observed asymmetry should not be interpreted as definitive evidence of publication bias

**Supplementary Figure S11. Trim-and-fill funnel plot for the MoCA outcome.**

A trim-and-fill sensitivity analysis was performed to assess the potential influence of missing studies on the pooled MoCA effect estimate. No potentially missing studies were imputed, and the pooled effect estimate remained unchanged (Hedges’ g = 1.69, 95% CI 1.15 to 2.22), suggesting that the pooled result was not materially altered by the trim-and-fill procedure.

**Supplementary Figure S12. Funnel plot for the MMSE outcome.**

The funnel plot is presented for descriptive purposes only because the MMSE meta-analysis included only five datasets, which is insufficient for reliable formal assessment of funnel-plot asymmetry or small-study effects. No formal test for small-study effects was performed.

**Supplementary Figure S13. Funnel plot for the RBMT outcome.**

The funnel plot is presented for descriptive purposes only because the RBMT meta-analysis included only three datasets, precluding reliable formal assessment of funnel-plot asymmetry or small-study effects. No formal test for small-study effects was performed.

**Supplementary Table S2. Study-level risk-of-bias assessment based on an adapted Cochrane RoB 1.0 framework**

| **Study** | **Random sequence generation** | **Allocation concealment** | **Blinding of participants and personnel** | **Incomplete outcome data** | **Selective reporting** | **Other bias** | **Overall risk of bias** |
| --- | --- | --- | --- | --- | --- | --- | --- |
| Zhu et al., 2020 | Low | Unclear | High | Low | Low | Unclear | High |
| Han et al., 2013 | Unclear | Unclear | High | Low | Low | Low | High |
| Zhang et al., 2021 | Low | Unclear | Unclear | Low | Low | Unclear | Unclear |
| Wen et al., 2018 | Unclear | Unclear | High | Low | Low | Unclear | High |
| Deng et al., 2019 | Low | Unclear | High | Low | Low | Unclear | High |
| Quan et al., 2025 | Low | Unclear | High | Low | Low | Unclear | High |
| Zhou et al., 2023 | Low | Unclear | High | Low | Low | Unclear | High |
| Sun et al., 2015 | Unclear | Unclear | High | Low | Low | Unclear | High |
| Liu et al., 2025 | Low | Unclear | Low | Low | Low | Low | Unclear |
| Lv et al., 2024 | Unclear | Unclear | Unclear | Low | High | Low | High |
| Chen et al., 2025 | Unclear | Unclear | Low | Unclear | Low | Unclear | Unclear |
| Gy et al., 2021 | Low | Low | Low | Low | Low | Low | Low |
| Riccitelli et al., 2025 | Unclear | Unclear | Low | Low | Low | Unclear | Unclear |
| Marra et al., 2015 | Low | Low | Low | Low | Low | Low | Low |

Note: Risk-of-bias assessments were based on six prespecified domains adapted from the Cochrane RoB 1.0 framework. Blinding of outcome assessment was not evaluated as a separate domain. Overall risk of bias was classified as Low when all six assessed domains were rated Low; High when at least one assessed domain was rated High; and Unclear when no domain was rated High but at least one domain was rated Unclear. RoB, risk of bias.
